# Effects of *Bacillus velezensis* FKM10 for Promoting the Growth of *Malus hupehensis* Rehd. and Inhibiting *Fusarium verticillioides*

**DOI:** 10.3389/fmicb.2019.02889

**Published:** 2020-01-10

**Authors:** Chengqiang Wang, Dongying Zhao, Guozhen Qi, Zhiquan Mao, Xiuna Hu, Binghai Du, Kai Liu, Yanqin Ding

**Affiliations:** ^1^Shandong Engineering Research Center of Plant-Microbial Restoration for Saline-Alkali Land, College of Life Sciences, Shandong Agricultural University, Tai’an, China; ^2^College of Horticulture Science and Engineering, Shandong Agricultural University, Tai’an, China

**Keywords:** PGPR, *Bacillus velezensis*, pot experiment, intertranscriptomic analysis, mechanism

## Abstract

*Bacillus velezensis* is a novel species of *Bacillus* that has been widely investigated and used because of its direct or indirect growth improvement effect for many plants. *B. velezensis* FKM10 was previously isolated from rhizosphere soil of apple trees and shows potential as a plant growth-promoting and biocontrol bacterium. In this study, strain FKM10 was verified to inhibit some fungal pathogens of soil-borne plant diseases, produce siderophores to absorb ferric iron for plants, and degrade proteins. Pot experiments showed that the application of strain FKM10 could directly promote the growth of *Malus hupehensis* Rehd. by increasing biomass, promoting the absorption of nutrients, improving soil fertility, changing the soil microbial community structure, and reducing fungal diversity. The results of this study provided a basis for using strain FKM10 to improve crop yield and overcome diseases of plants. The mechanism of strain FKM10 to control the phytopathogenic fungus *Fusarium verticillioides* was studied by interoperation with RNA sequencing. Strain FKM10 can destroy the cell wall and cell membrane of *F. verticillioides*. The secretion of glucosidases, such as β-glucanase, might be one of the causes of the destruction of the fungal cell wall. The regulation of amino acid metabolism might also play an important role in the antibacterial process of strain FKM10. During the antibacterial process, strain FKM10 attacks *F. verticillioides* and strain FKM10 itself is also affected: the expression of spores is increased, the number of viable cells is decreased, and the ribonucleoprotein complex and flagellar assembly-related genes are downregulated. The results of this study indicate that both strain FKM10 and *F. verticillioides* have mutually inhibitory activities in a liquid environment. Comparative genome analysis of *B. velezensis* FKM10 reveals that the general features of their genomes are similar overall and contain the core genome for this species. The results of this study further reveal that *B. velezensis* can also serve as a basis for developing new biocontrol agents or microbial fertilizers.

## Introduction

Plant growth-promoting rhizobacteria (PGPR) possess functions of improving plant growth by inhibiting plant pathogens, producing phytohormones, producing siderophores, solubilizing phosphate, and fixing nitrogen, for example (Beneduzi et al., 2012; Bhattacharyya and Jha, 2012; Bulgarelli et al., 2013). One of the most important applications of PGPR is defending the plant from pathogenic or parasitic organisms by direct or indirect inhibition, such as competing nutrients or producing antimicrobial compounds (Kim et al., 2011; Eastman et al., 2014). In agriculture, PGPR has the capability and value to replace some chemical fertilizers and pesticides (Bhattacharyya and Jha, 2012). Therefore, identifying a variety of PGPR is meaningful.

Many plant growth-promoting and biocontrol *Bacillus* strains have been recently reported, including *Bacillus subtilis*, *Bacillus amyloliquefaciens*, and *Bacillus velezensis*. *B. velezensis* is a widespread Gram-positive bacterium, which was named in 2005 by Ruiz-García et al. (2005), and it has later heterotypic synonyms: *Bacillus methylotrophicus* (Liu et al., 2018), *B. amyloliquefaciens* subsp. *Plantarum* (Pinto et al., 2018), and “*Bacillus oryziocola*” (Dunlap et al., 2016). They belong to PGPR and could be used in agriculture (Chen et al., 2007; Jeukens et al., 2015), for example, to promote plant growth and control pathogenic microorganisms by producing some secondary metabolites or antibiotics, and efficient colonization of plants (Adeniji and Babalola, 2019). Auxin secretion by *B. velezensis* FZB42 could increase the exudation of organic carbon and promote root growth for *Triticum aestivum* (Talboys et al., 2014). *B. velezensis* GH1-13 was reported to benefit plant growth by nutrient uptake and secreting secondary metabolites such as asindole-3-acetic acid to promote the system development of plant roots (Kim et al., 2017). *B. velezensis* BAC03 promoted plant growth by secreting several substances such as asindole-3-acetic acid and ammonia (Meng et al., 2016). *B. velezensis* CBMB205, a phosphate-solubilizing bacterium isolated from the rhizosphere soil of rice, was also reported to efficiently promote plant growth (Hwangbo et al., 2016). *B. velezensis* could also cowork with other PGPR; for example, *B. velezensis* S141 coinoculated with *Bradyrhizobium diazoefficiens* USDA110 could increase soybean growth, nodulation, and N_2_ fixation efficiency (Sibponkrung et al., 2017). To date, *B. velezensis* was reported to inhibit the growth of many pathogenic fungi, such as *Cryphonectria parasitica* (Xu et al., 2016), *Helicobasidium purpureum* (Xu et al., 2016), *Cylindrocladium quinqueseptatum* (Xu et al., 2016), *Ralstonia solanacearum* (Cao et al., 2018), *Fusarium oxysporum* (Cao et al., 2018), *Aspergillus flavus* (Chen et al., 2019), and Rhizoctonia *solani* AG1-IB (Chowdhury et al., 2013), by the biosynthesis of β-1,3-1,4-glucanase, lipopeptide antibiotics (surfactin, iturin, and fengycin, for example), polyketides (actinomycin D, bacitracin, and cyclosporin A, for example), and iron carriers (Stein, 2005; Chen et al., 2007; Meng et al., 2016; Kim et al., 2017; Fan et al., 2018). *B. velezensis* can also trigger systemic resistance in plants (Rabbee et al., 2019). Many studies of this species were focused on its properties and application to improve plant growth and control fungal pathogens of plant disease. However, the mechanisms of *B. velezensis* for promoting plant growth and inhibiting fungal pathogens remain basically clear.

*Bacillus velezensis* FKM10 was formerly isolated by our group from the rhizosphere soil of apple trees in Shandong, China. Strain FKM10 was tested to promote plant growth and inhibit some pathogenic fungi. The characteristics of strain FKM10 for promoting the growth of *Malus hupehensis* Rhed., a valuable and scarce rootstock resource of apple trees in China, were performed through pot experiments. At the same time, the mechanism of strain FKM10 for inhibiting the growth of pathogenic *Fusarium verticillioides* was also revealed by intertranscriptomic analysis. Genomic comparison further elaborated the genomic and functional conservation of *B. velezensis* species.

## Materials and Methods

### Determination of Biological Traits

#### Analysis of Degrading Protein

The capacity of degrading protein was tested on medium containing 1.5% (w/v) skim milk powder and 1.6% (w/v) agar at pH 7.0–7.2. Strains were inoculated and cultured at 37°C for 2 days to observe the transparent region produced by degrading the skim milk powder.

#### Analysis of Antagonistic Activities (Du et al., 2019)

The pathogenic hyphae were taken as a rondure of 6-mm diameter and then placed on the center of a potato dextrose agar (PDA) plate. The plate was then incubated for 2 days at 28°C. Strains were then streaked at a distance of 1 cm from the edge of the pathogenic fungal hyphae and cultured at 28°C for another 2 days. The inhibition zones were measured to judge the antagonistic activities.

#### Qualitative Analysis of Siderophores

Clones were cultivated overnight on LB plates at 37°C. The clones were then inoculated on a CAS-agar plate for qualitative analysis of siderophores (Guo et al., 2016). The yellow circles appeared around the colonies were measured (Schwyn and Neilands, 1987).

#### β-Glucanase Activity Analysis (Xu et al., 2016)

Strains were inoculated on the glucanase identification medium (0.2% β-glucan, 0.2% NaNO_3_, 0.1% K_2_HPO_4_, 0.05% KCl, 0.05% MgSO_4_, 0.001% FeSO_4_, 0.005% Congo red, 2% agar, pH 7.2) and incubated at 37°C for 3 days. The transparent circles around the colonies were observed.

### Pot Experiment

The pot experiment of *M. hupehensis* Rehd. was begun on May 8. The strain FKM10 was applied on May 27 and plants were harvested on October 18, for a total of 163 days of growth. Pots 16 cm in height and 25 cm in diameter were used per pot with 4 kg continuous cropping soil of apple tree. The seedlings of *M. hupehensis* Rehd. were planted and watered according to the weather and other conditions, and managements such as deworming, spraying, and loosening were carried out according to the growth conditions. Two treatments were set up with 15 replicates per treatment. The seedlings of *M. hupehensis* Rehd. with five leaves and one core, which have the same growth status, were selected and transplanted into new pots, one plant per pot. The transplanted seedlings were first treated with strain FKM10 after 10 days. The amount of strain FKM10 applied per pot: 8 ml (2 × 10^9^ CFU/ml) of the bacterial suspension with bean juice culture was added to 800 ml of clear water and poured in the rhizosphere of *M. hupehensis* Rehd. The seedlings were treated a second time with strain FKM10 after the first application for 70 days. The control treatment was applied in the same volume of bean juice culture without the bacteria FKM10.

The agronomic traits of *M. hupehensis* Rehd., including plant height, stems, and leaves, were measured every 10 days after 30 days of the first application of strain FKM10. Determination of active iron in the leaves of *M. hupehensis* Rehd.: 0.1 g of dried leaves (less than 0.3 mm) was collected and placed in 25 ml of 1 mol/ml hydrochloric acid, kept at room temperature for 24 h, and shaken three to four times. After filtration, the extract was tested by atomic absorption spectrophotometry, as reported (Pierson and Clark, 1984). The leaves of *M. hupehensis* Rehd. were also collected at 30, 45, 60, 75, and 90 days after the application of strain FKM10. A total of 0.5 g leaves were placed in a mortar and ground with a small amount of liquid nitrogen. A total of 5 ml extract liquid (precooled at 4°C) and a small amount of quartz sand were added and then centrifuged at 12,000 *g*/min for 20 min at 4°C. The supernatant was the crude enzyme solution that was used for testing the superoxide dismutase (SOD) and peroxidase (POD) enzyme activities by the method of Farhangi-Abriz and Torabian (2017). Determination of chlorophyll: 0.1 g of the top leaves of apple seedlings (the same leaf position taken for each treatment) was collected, immersed in 95% ethanol, and then placed in the dark for 40 h, during which it was shaken five to six times. The extracted liquids were taken to measure the absorbance at 663 and 645 nm (Palta, 1990). Determination of the biomass of *M. hupehensis* Rehd.: the potted plants were collected after the application of strain FKM10 for ∼140 days, washed with deionized water, and then measured for the fresh weight. After drying, the dry weight of *M. hupehensis* Rehd. was also measured. The total amounts of nitrogen, phosphorus, and potassium contents of *M. hupehensis* Rehd. were extracted for decoction by the H_2_SO_4_-H_2_O_2_ digestion method (Bowman, 1988). The total phosphorus was then determined by the vanadium-molybdenum yellow colorimetric method (Ku et al., 2018), the total potassium was determined by the flame photometry method (Ahamed and Abdalla, 2019), and the total nitrogen was determined by a flow analyzer (Schuman et al., 1973).

Determination of available iron in soil: The air-dried soil from each treatment was extracted by DTPA, and the content of available iron in the soil was determined by atomic absorption spectrophotometry (mg/kg) as reported (Pierson and Clark, 1984). Determination of soil enzyme activity: (1) Urease activities were determined by a colorimetric method. The enzyme activities were shown by the mass (mg) of NH3-N in 1 g soil after 24 h. (2) Phosphatase activities were determined by the colorimetric method of phenylphosphonate. The mass (mg) of phenol indicated the enzyme activities. (3) Soil invertase activities were determined by the colorimetric method of 3,5-dinitrosalicylic acid, and the amounts of produced glucose in 1 g dry soil after 24 h showed the invertase activities. (4) Soil catalase activities were determined by potassium permanganate titration. The enzyme activities were determined as the consumed volume of 0.02 mol/l KMnO_4_ per 1 g dry soil in 1 h (Sun et al., 2017). The soil available nutrient contents: the soil available phosphorus was extracted with 0.5 mol/l sodium bicarbonate-molybdenum antimony colorimetric method; the soil available potassium was diluted with 1 mol/l ammonium acetate Lift-flame photometer method; and the soil available nitrogen was extracted by a calcium chloride-flow injection analyzer (Yu et al., 2015).

At the end of the pot experiments, the rhizosphere soil that closely attached to the root surface of *M. hupehensis* Rehd. was collected and the total DNA of the soil genome was extracted using the E. Z. N. A. Soil DNA Kit (Omega Bio-tek, Norcross, GA, United States). The 16S rDNA V4 region-specific primers B341F (5′-CCTACGGGNGGCWGCAG-3′) and B785R (5′-GACTACHVGGGTATCTAATCC-3′) (Klindworth et al., 2013) were used to amplify the 16S rDNA sequences. The ITS1 region-specific primers EF4 (GGAAGGGRTGTATTTATTAG) and fung5 (GTAAAAGTCCTGGTTCCCC) (Smit et al., 1999) were used to amplify the sequences of ITS1 rDNA. The DNA samples were sent to Shanghai Paisen Bioengineering Co., Ltd., for sequencing and then analyzed by Illumina MiSeq sequencing. Clean reads were obtained when the raw data were filtered to eliminate the reads with sequencing adapters, ambiguous N base, poly base, or average base quality scores less than 20. Using USEARCH and UPARSE (Edgar, 2013), the sequences were clustered to operational taxonomic unit (OTU) at 97% sequence similarity and analyzed. The mothur or R software is used to generate the Venn diagram of the inter-sample (or inter-group) OTU, and the Alpha diversity index is calculated using mothur (Schloss et al., 2009).

### Strain FKM10 Interacts With *F. verticillioides*

Strain FKM10 and *F. verticillioides* were inoculated into PDA liquid medium and cultured for 12 h at 28°C. Strain FKM10 was then inoculated in 20 ml PDA liquid medium with 1% inoculation; meanwhile, *F. verticillioides* was inoculated into 40 ml of PDA liquid medium with 1.5% inoculation for 10 h at 28°C. For the treatment group (DT), where strain FKM10 and *F. verticillioides* were inoculated together, 20 ml culture of strain FKM10 was mixed with 40 ml culture of *F. verticillioides*. For the bacterial control group (FKM10), 20 ml culture of strain FKM10 was mixed with 40 ml PDA liquid medium. For the fungal control group (FV), 20 ml PDA liquid medium was mixed with 40 ml culture of *F. verticillioides*. After coculture for 3 h, the samples underwent intertranscriptomic analysis.

### Total RNA Extraction and Intertranscriptomic Analysis

Total RNA was extracted from tissue samples using the TRIzol^®^ Reagent (Invitrogen, Carlsbad, CA, United States) following the manufacturer’s protocol. The concentration and purity of extracted RNA were detected using Nanodrop 2000 spectrophotometer, RNA integrity was evaluated by agarose gel electrophoresis, and RNA Integrity Number was determined by Agilent 2100 (Agilent, Santa Clara, CA, United States). The samples with RNA Integrity Number ≥ 7 were subjected to the following analysis. For strain FKM10, rRNA was removed by Ribo-Zero Magnetic kit (EpiCentre, Madison, WI, United States) and mRNA was obtained. For *F. verticillioides*, using beads with Oligo (dT) to pair with ployA for A-T pairing, mRNA can be obtained from total RNA. Qualified RNA was further purified by Certified Low Range Ultra Agarose (Bio-Rad, Hercules, CA, United States). The fragmentation buffer was then added to break the mRNA into approximately 200-bp fragments. RNA-Seq libraries were prepared by Truseq^TM^ RNA sample prep Kit and sequenced using Illumina HiSeq 4000 sequencer (Illumina Inc., San Diego, CA, United States).

After sequencing, 150/300-bp paired-end reads were generated. Raw data were filtered to obtain high-quality sequencing data (clean data). The reads containing ploy-N and the low-quality reads were removed, and then the clean reads were obtained^1^. Then the clean reads were mapped to the reference genome using Bowtie2^2^. Identification of differentially expressed gene (DEG) and annotation: FPKM (fragments per kilobase of exon per million fragments mapped) (Trapnell et al., 2010) of each gene was calculated using RSEM^3^. DEGs were identified using the edger^4^. | log2FC| ≥ 1 and *p*-value < 0.05 were set as the threshold for significantly differential expression. Kyoto Encyclopedia of Genes and Genomes (KEGG^5^) pathway enrichment analysis of DEGs was performed using KOBAS^6^.

### Determine Spores and Viable Cells

The cells were taken at different time points for dilution and then placed at 80°C for 15 min to kill the viable cells. The mature spores survived due to good heat resistance. The treated samples were inoculated on LB plates to cultivate at 37°C. The number of viable cells was also determined at different time points for dilution and then inoculated on LB plates to cultivate at 37°C. The formed colonies were calculated to determine the number of spores and viable cells.

### Analysis of Core and Specific Genes

Core/Pan genes of strain FKM10 with strains FZB42, JJ-D34, YJ11-1-4, and JS25R were clustered by the CD-HIT (Li and Godzik, 2006) rapid clustering of similar proteins software with a threshold of 50% pairwise identity and 0.7 length difference cutoff in amino acids. The core genes are homologous genes that contain all strains. The dispensable genes are obtained by removing the common genes. All the dispensable genes combined with the core genes are pan genes. Specific genes are the ones owned by only one strain.

### COG Functional Categorization

The clusters of orthologous groups (COGs) (Tatusov et al., 1997, 2003) were determined according to the National Center for Biotechnology Information^7^. Using BLAST software, protein sequences were blasted and then annotated into different COGs, which were consisted by orthologous sequences and could predict the function of protein sequences. As the alignment results of each sequence might be more than one, the gene annotation was retained by the optimal matching result.

### Statistical Analysis

The data were analyzed using ANOVA and Duncan’s multiple-range test (*p* ≤ 0.05) using statistical software SPSS version 19.0 (SPSS Inc., Chicago, IL, United States). The significance between the means of different treatments was evaluated using Duncan’s (D) test (Tahir et al., 2017).

## Results

### General Characteristics of Strain FKM10 Identified as PGPR

**FIGURE 1:**
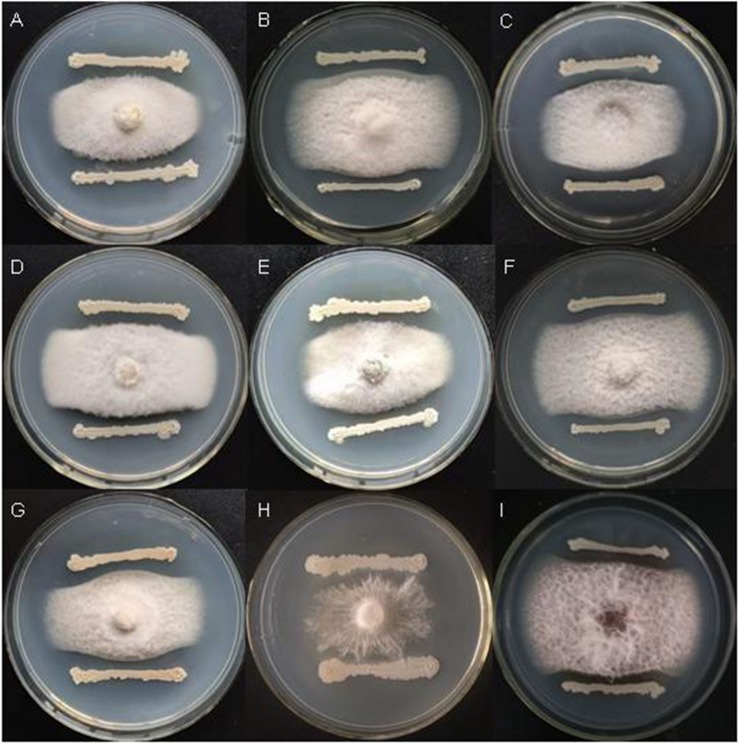
Strain FKM10 inhibits some pathogenic fungi of soil-borne plant diseases. **(A)** *Botrytis cinerea*, **(B)** *Fusarium Solani*, **(C)** *F. proliferatum*, **(D)** *F. vasinfectum*, **(E)** *F. solani (Mart.) Sacc.*, **(F)** *F. oxysporum*, **(G)** *F. sp.*, **(H)** *Sclerotium rolfsii Sacc.*, and **(I)** *F. verticillioides*. The preserved pathogenic fungus hyphae were placed in the center of PDA plates. The plates were incubated for 2 days in a 28°C incubator. FKM10 was then streaked at a distance of 1 cm from the edge of the pathogenic fungus hyphae and cultured at 28°C for another 2 days.

**FIGURE 2:**
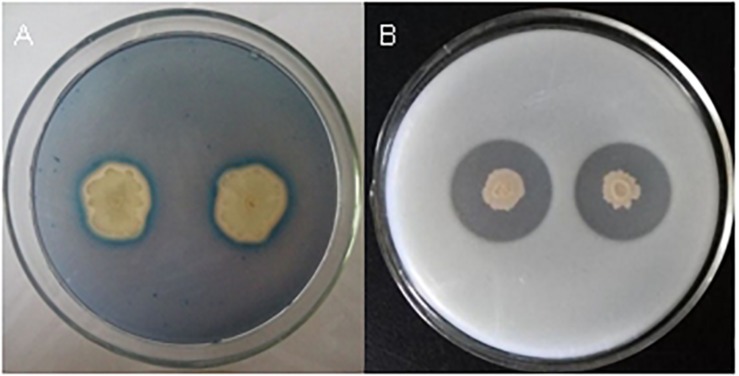
Tests of siderophores and protease production capacity of strain FKM10. **(A)** Qualitative analysis of siderophores. Single clones of strain FKM10 were cultivated on LB plates overnight at 37°C. The bacterial lawn was then inoculated on a CAS-agar plate for qualitative analysis of siderophores. **(B)** Protease production capacity test. Strain FKM10 was inoculated on a skim milk powder medium and cultured at 37°C for 2 days to yield a transparent circle generated around the colonies.

*Bacillus velezensis* FKM10 (formerly named *B. methylotrophicus* and now reclassified as *B. velezensis*) was isolated by our group from an apple rhizosphere in Shandong, China. The whole-genome sequence of strain FKM10 was also obtained (Wang et al., 2016) and uploaded to GenBank (accession number LNTG00000000). Strain FKM10 was verified here to inhibit some fungal pathogens of soil-borne plant diseases (Figure 1), to produce siderophores to absorb ferric iron, and to degrade protein (Figure 2). These features of strain FKM10 indicated that it could act as an important plant growth-promoting rhizobacterium.

### Strain FKM10 Promotes the Growth of *M. hupehensis* Rehd.

#### Effects of Strain FKM10 on Agronomic Characteristics of *M. hupehensis* Rehd.

*Malus hupehensis* Rehd. is a valuable and scarce rootstock resource for apple trees in China (Benson et al., 2001; Lin and Sun, 2017). Moreover, the roots of *M. hupehensis* Rehd. are sensitive to soil environmental changes, and there is a significant correlation between plant shoot variation and root variation, which has important research and utilization value in apple rootstock breeding and production (Yang et al., 2008; Yang and Fan, 2012).

**FIGURE 3:**
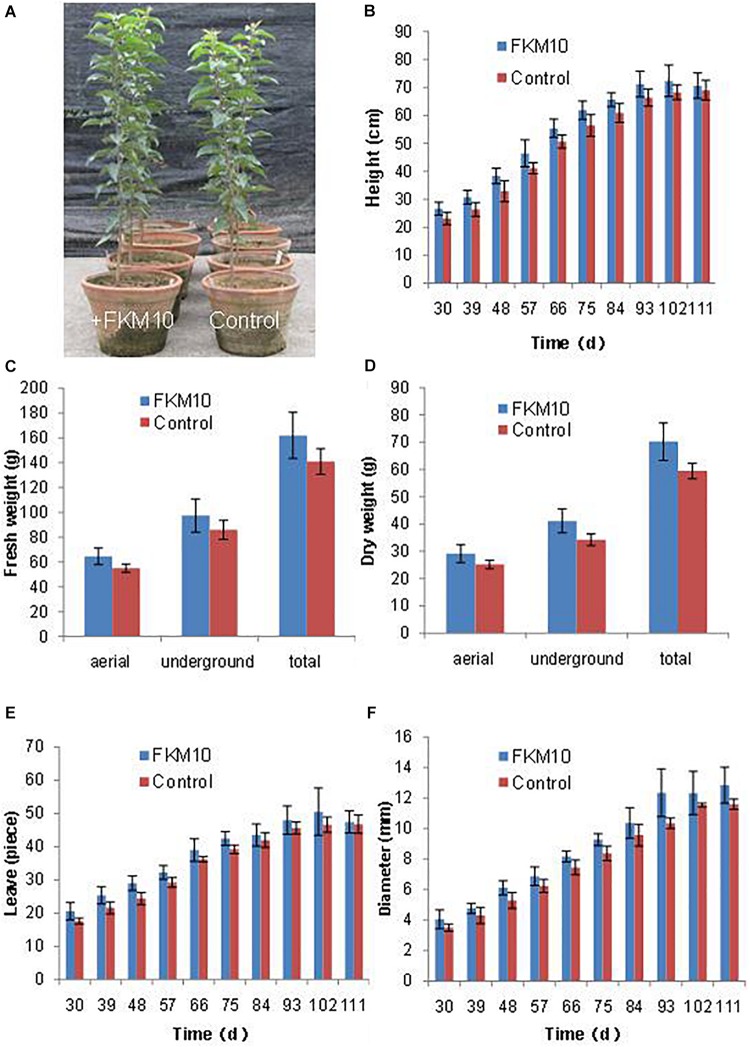
The agronomic characteristics of *M. hupehensis* Rehd. The amount of strain FKM10 applied: 8 ml (2 × 10^9^ CFU/ml) of the bacterial suspension (with bean juice culture) was added to 800 ml of clear water and poured in the rhizosphereof *M. hupehensis* Rehd. The control group was treated with the same volume of bean juice culture without bacteria. The final potted effect of *M. hupehensis* Rehd. was observed **(A)**. The agronomic traits of *M. hupehensis* Rehd. were determined every 10 days after the first application of strain FKM10 for 30 days: plant height **(B)**, number of leaves **(C)**, and diameter of stems **(D)**. After growth for 163 days, the roots of *M. hupehensis* Rehd. were washed with water and dried with absorbent papers to determine the fresh weight of the aerial part and the underground part of *M. hupehensis* Rehd. **(E)**. The plants were then treated at 105°C for 30 min and dried at 60°C to constant weight, and the dry weights were measured **(F)**. A total of two treatments were set up in the pot experiments with 15 replicates for each treatment. Error bars indicate the SD from the mean.

*Bacillus velezensis* FKM10 could significantly promote the growth of *M. hupehensis* Rehd. In pot experiments, the seedlings of *M. hupehensis* Rehd. were cultivated to grow, transplanted, and then treated with strain FKM10. The plants were harvested after a total growth of 163 days. The final plant chart is shown in Figure 3A. The height, basal diameter, and number of leaves of *M. hupehensis* Rehd. at different growth periods after the application of strain FKM10 were determined (Figures 3B–F, respectively). After strain FKM10 application for 30 days, the agronomic traits of the treated group were better than those of the control group. From 30 to 66 days after strain FKM10 application, the plants of the treatment group were significantly higher than the control group (*p* < 0.05). The number of leaves in the treatment group was also more than that of the control group at 30, 39, 48, 57, and 75 days after the application of strain FKM10 (*p* < 0.05), increasing by 17.05, 17.59, 18.85, 10.45, and 8.42%, respectively. At 48, 66, 75, and 93 days after strain FKM10 application, the stem diameters of the treated group were significantly different from that of the control group (*p* < 0.05), increasing by 15.56, 9.68, 10.80, and 19.39%, respectively. Strain FKM10 can promote the growth of the aerial parts of *M. hupehensis* Rehd. at the test growth stages. After growth for 163 days, the roots of *M. hupehensis* Rehd. were washed with water and dried with absorbent papers to determine the fresh weight of the aerial part and the underground part. The results showed that the fresh weight of the treated group was higher than that of the control group, as shown in Figure 3C. However, only the fresh weight of the aerial part reached a significant difference (*p* < 0.05) compared with the control group, which was increased by 17.1%. The plants were then treated at 105°C for 30 min, then dried at 60°C to constant weight, and the dry weight was measured (Figure 3D). The dry weight of the treated group and control group reached a significant difference (*p* < 0.05), where the dry weights of the aerial part, underground part, and total plant were increased by 15.87, 19.94, and 18.20%, respectively. These results indicated that strain FKM10 can significantly increase the biomass of *M. hupehensis* Rehd.

#### Effects of Strain FKM10 on Active Components of *M. hupehensis* Rehd.

**FIGURE 4:**
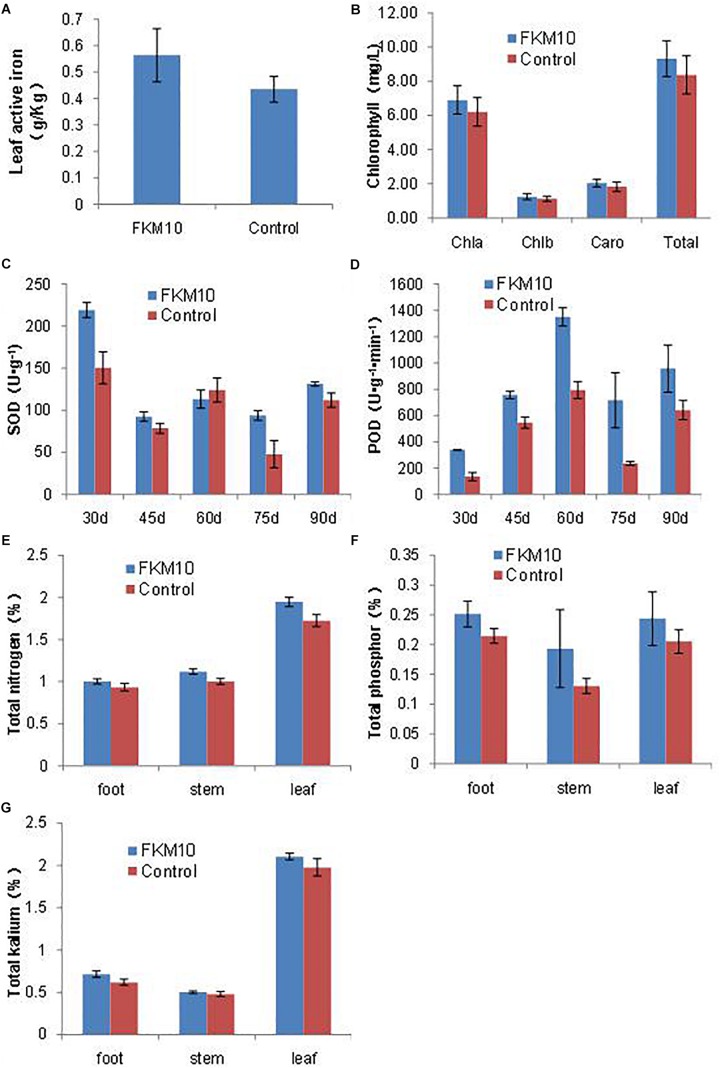
Active components of *M. hupehensis* Rehd. The active iron **(A)** and chlorophyll **(B)** contents of leaves. Chlorophyll was extracted by 95% ethanol for 40 h in the dark, and the extract was measured at 663 and 645 nm. The activity of superoxide dismutase (SOD) **(C)** and peroxidase (POD) **(D)** of leaves. The SOD activities of leaves were determined every 15 days after the application of strain FKM10 for 30 days. The contents of total nitrogen **(E)**, phosphorus **(F)**, and potassium **(G)** in different parts of *M. hupehensis* Rehd. Error bars indicate the SD from the mean.

Iron is essential for chlorophyll synthesis and some enzyme activities. The active iron content of leaves is a reference index to iron nutrition in plants. The application of strain FKM10 could significantly increase the active iron content of leaves, which contributes to the synthesis of chlorophyll and promotes the growth of the plant. After the application of strain FKM10, the content of active iron in *M. hupehensis* Rehd. leaves was increased by 29.26%, reaching a significant difference compared with the control group (*p* < 0.01) (Figure 4A). Iron deficiency can cause yellowing in plants and seriously reduces the synthesis of chlorophyll. Although the chlorophyll contents of plants were also increased after the application of strain FKM10 (Figure 4B), they did not reach a significant difference.

After treatment with strain FKM10 for 30, 45, 60, 75, and 90 days, functional leaves were collected and their SOD and POD activities were measured. The SOD activities of leaves in the treated group were significantly different from those in the control group at 30, 45, 75, and 90 days after the application of strain FKM10 (Figure 4C), increasing by 45.6, 17.47, 96.83, and 17.20%, respectively. At 30, 45, 60, 75, and 90 days, the POD activities of the treated group and the control group reached a significant difference, increasing by 150.53, 38.76, 69.93, 204.97, and 69.95% (Figure 4D), respectively. The results showed that strain FKM10 could stably induce the systemic resistance of *M. hupehensis* Rehd. for a long time and improve the stress resistance, which benefits the growth of *M. hupehensis* Rehd.

The contents of total nitrogen, phosphorus, and potassium in the roots, stems, and leaves of *M. hupehensis* Rehd. were determined (Figures 4E–G, respectively). The nitrogen contents of roots, stems, and leaves in the treated group were higher than those in the control group, increasing by 7.22, 11.63, and 12.86%, respectively. After strain FKM10 application, the contents of total phosphorus in roots and stems in the treated group were increased compared to the control by 17.30 and 48.07%, respectively. The contents of potassium in roots and leaves of the treated group were increased by 15.31 and 6.53%, respectively, compared to the control. Strain FKM10 could promote the accumulation of nitrogen, phosphorus, and potassium in *M. hupehensis* Rehd.

#### Effects of Strain FKM10 on the Features of the Rhizosphere Soil of *M. hupehensis* Rehd.

Soil enzymes involved in biochemical processes and chemical reactions in the soil are closely related to the cycle of mineral elements, the transfer of energy, and the environmental quality. Soil enzyme activities can reflect not only the level of microbial activities in the soil but also the ability of soil nutrient transformation and transfer, which is one of the important indexes for evaluating soil fertility (Marx et al., 2001; Vepsäläinen et al., 2001; Boerner et al., 2005). Strain FKM10 can significantly affect the rhizosphere soil properties. The activities of urease, phosphatase, sucrose, and catalase in the rhizosphere soil of *M. hupehensis* Rehd. were measured (Table 1). The activities of soil urease, phosphatase, and sucrase in the treated group were all higher than those in the control group, increasing by 35.08, 19.72, and 36.26%, respectively. However, the catalase activities were lower. Soil catalase activity is related to the soil respiration intensity and microbial activity, which is a reflection of microbial activity intensity. The reduced catalase activity in the treated group might be because strain FKM10 can antagonize fungi and reduce the richness and diversity of fungi in the soil.

**TABLE 1:** Properties of the rhizosphere soil of *M. hupehensis* Rehd. after the application of strain FKM10.

		**Control**	**FKM10**
The enzyme activities of soil	Phosphatase mg/(g⋅24 h)	24.73 ± 3.70	29.60 ± 2.39^∗∗^
	Urease NH3-H mg/(g⋅24 h)	1.29 ± 0.08	1.75 ± 0.05^∗∗^
	Sucrase mg/(g⋅24 h)	9.43 ± 0.79	12.85 ± 1.04^∗^
	Catalase (0.1 mol/l KMnO4) (g⋅h)	2.68 ± 0.16	2.29 ± 0.13
The nutrient	Nitrate nitrogen	9.82 ± 0.81	11.25 ± 0.60
contents of soil	Ammonium nitrogen	3.07 ± 0.62	4.27 ± 0.91
	Available phosphorus	60.35 ± 6.69	65.36 ± 4.89
	Available kalium	205.58 ± 4.58	196.10 ± 10.05
	Available iron	28.50 ± 1.18	36.76 ± 3.63^∗^

*Values are mean ± SD. “^∗∗^” and “^∗^” indicate the differences of FKM10 application group compared to the control group at *p* < 0.01 and *p* < 0.05, respectively.*

The amounts of ammonium and nitrate nitrogen in soil can reflect the nitrogen supply of the soil. After the application of strain FKM10, the contents of ammonium and nitrate nitrogen in the treatment group were higher than those in the control group, increasing by 14.6 and 39.28%, respectively (Table 1). Soil available phosphorus refers to the water-soluble and easily converted phosphorus among the total phosphorus in the soil, which has a close correlation with the amount of phosphorus absorbed by plants from the soil. Soil available potassium refers to the potassium that plants can absorb and utilize in a short time. The content of available iron in rhizosphere soil can reflect the actually available iron for plants. Compared with the control group, available phosphorus and potassium showed no significant changes in the treatment group, but available iron in the soil was significantly increased by 28.98% (Table 1).

#### Effects of Strain FKM10 on the Microbial Communities of the Rhizosphere Soil of *M. hupehensis* Rehd.

**FIGURE 5:**
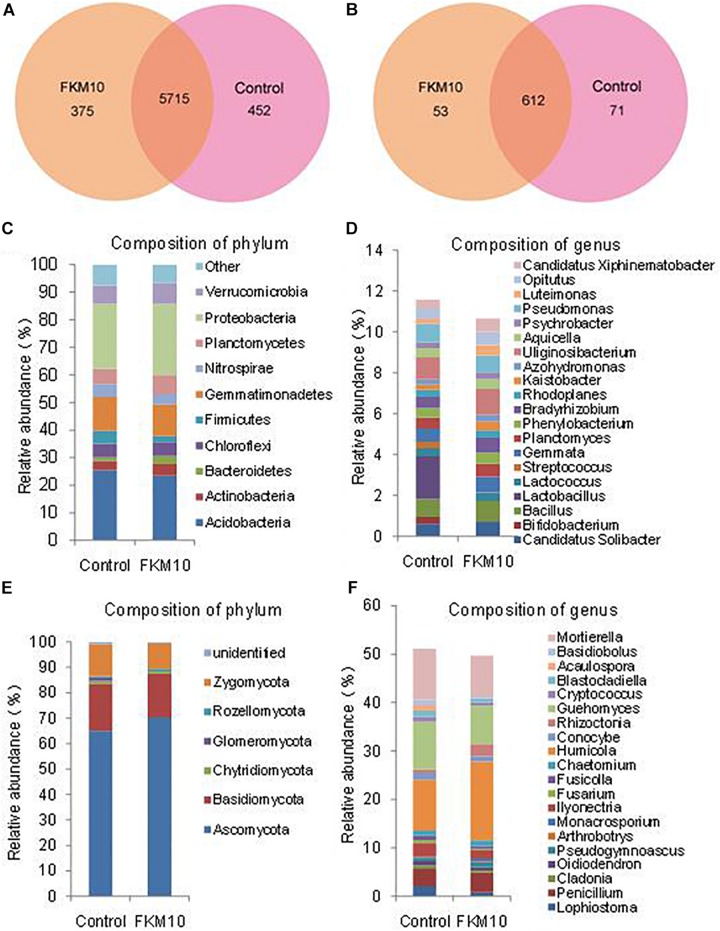
Composition and relative abundance of major bacterial phyla and genera. Venn analysis of OTU in different treatments based on 16S rDNA **(A)** and ITS1 **(B)**. Composition and relative abundance of different bacterial **(C**,**D)** and fungal **(E**,**F)** community structures at the phylum and genus levels (the top 20).

The microbial community structures of rhizosphere soil of the treated group and the control group were studied by Illumina sequencing. The sequence information and the calculated index of microbial diversity are listed in Supplementary Table S1. Compared with the control group, the ACE and Chao index of bacteria and fungi were all increased in the treated group. The Shannon index of the treated group was higher compared with the control group in the bacterial community but was lower in the fungal community. The results indicated that the diversity of bacteria was increased and the diversity of fungi was reduced after the application of strain FKM10. The Venn diagram was used to evaluate the distribution of OTUs in different treatments. In the bacterial community (Figure 5A), a total of 5715 OTUs (containing 87.36% of the sequences) were common, 375 OTUs were specific for the treated group, and the control group had 452 unique OTUs. In the fungal community (Figure 5B), a total of 612 OTUs (containing 83.15% of the sequences) were common, 53 OTUs were specific for the treated group, and the control group had 71 unique OTUs.

Bacteria from the treated and control groups showed different abundances. The relative abundance of bacterial communities at the phylum level is shown in Figure 5C. A total of 21 identified phyla were obtained; Acidobacteria, Proteobacteria, and Gemmatimonadetes were the dominant phyla in the two groups. The relative abundances of phyla, especially for the main phyla, were changed by the application of strain FKM10. In the control group, Acidobacteria was 25.44% of the total bacteria, while the percentage in the treatment group was reduced to 23.50%. Bacteroidetes were significantly enriched in the treated group, accounting for 3.00%, compared with 1.28% in the control group. The relative abundance of bacterial communities at the genus level was further analyzed (Figure 5D). A total of 246 genera were classified among all the soil samples. The genera *Uliginosibacterium*, *Lactobacillus*, *Pseudomonas*, *Bacillus*, and *Gemmata* were dominant. Compared with the control group, *Bifidobacterium*, *Lactobacillus*, and *Streptococcus* were drastically reduced in the treated group, while *Bradyrhizobium*, *Kaistobacter*, and *Luteimonas* were significantly increased.

The relative abundance of the fungal communities at the phylum level is shown in Figure 5E. There were six classified phyla; Ascomycota, Basidicota, and Zygomycota were the dominant phyla. In the control group, Ascomycota was 65.00% of the total fungi and was enriched to 70.28% in the treatment group. The phylum Rozellomycota was 0.45% of the total fungi in the control group and was increased to 0.83% in the treatment group. Further analysis was conducted to illustrate the relative abundance of fungal communities at the genus level (Figure 5F). A total of 173 genera were classified among all samples. *Humicola*, *Mortierella*, *Guehomyces*, *Penicillium*, and *Ilyonectria* were the dominant genera. Compared with the control group, *Basidiobolus*, *Acaulospora*, and *Lophiostomas* were drastically reduced in the treated group, while *Humicola*, *Rhizoctonia*, and *Monacrosporium* were significantly increased.

The application of strain FKM10 can directly promote the growth of *M. hupehensis* Rehd. and optimize the properties of the rhizosphere soil (Table 1), reflecting the direct promoting function of strain FKM10. Meanwhile, strain FKM10 can inhibit fungal pathogens of soil-borne plant diseases (Figure 1) and lead to the decreased diversity of soil (Figures 5E,F), which can reduce plant diseases and indirectly promote plant growth.

### The Intertranscriptomic Analysis of FKM10 Inhibiting the Growth of *F. verticillioides*

**FIGURE 6:**
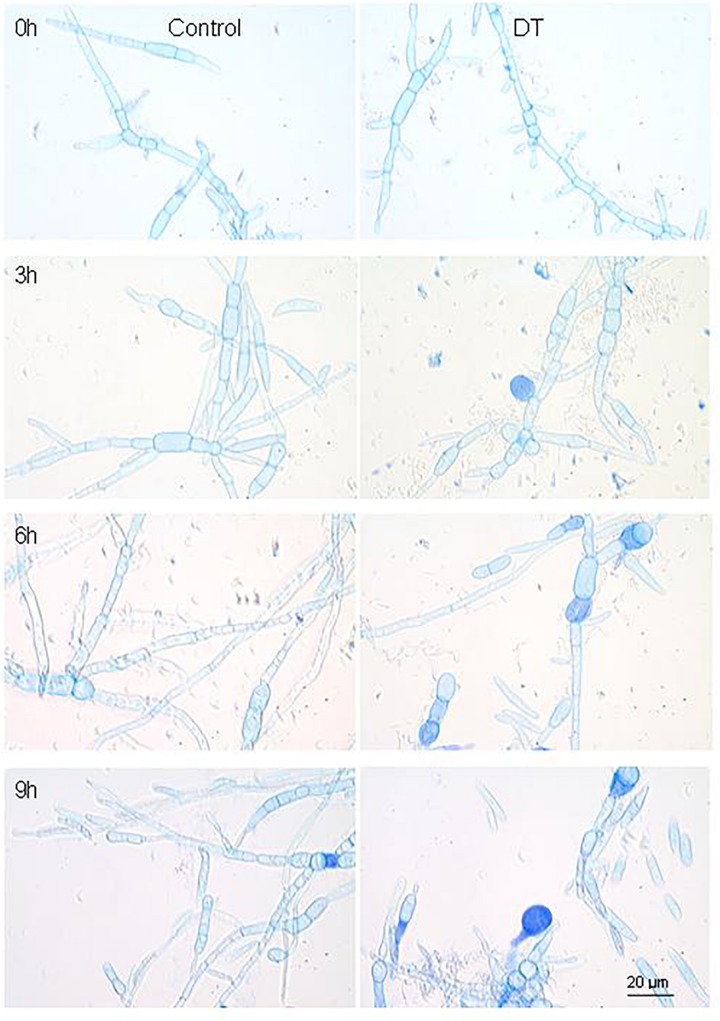
The mycelium morphology of *F. verticillioides* that was cocultivated with or without strain FKM10. “Control” was the group that was only cultivating *F. verticillioides*. “DT” was the group that was cultivating *F. verticillioides* with strain FKM10. Samples were taken at 3, 6, and 9 h after strain FKM10 and *F. verticillioides* were cocultivated. After staining with 0.4% trypan blue, the mycelium morphology was observed by microscopy using an OLYMPUS biological microscope at 1000×.

The transcriptome analysis of the antagonistic interaction between strain FKM10 and fungi can further elucidate the gene regulatory mechanism of strain FKM10 on pathogenic fungi. *F. verticillioides* is a common plant pathogen and strain FKM10 could effectively inhibit the growth of *F. verticillioides* (Figure 1). The mycelium condition of *F. verticillioides* could be observed by trypan blue staining, commonly used to test the integrity of cell membranes (Zhi et al., 2013). When strain FKM10 and *F. verticillioides* were cocultivated for 3 h, some of the mycelia of *F. verticillioides* were dyed blue (Figure 6). This result indicates that the cell membrane of *F. verticillioides* has been damaged. With time, more and more mycelia of *F. verticillioides* were dyed blue, and the damage to the cell membrane was more serious. Thus, we performed intertranscriptomic analysis by the coculture of strain FKM10 and *F. verticillioides* for 3 h.

To investigate the gene expression profiles of strains FKM10 and *F. verticillioides* when they were cocultivated, intertranscriptomic analysis was performed. A total of 375 genes of strain FKM10 were significantly different under cocultivated conditions, among which 87 genes were upregulated and 288 genes were downregulated (Supplementary Table S2). A total of 792 genes in *F. verticillioides* were significantly different under cocultivated conditions, among which 535 genes were upregulated and 257 genes were downregulated (Supplementary Table S3). To identify the functions of DEGs during coculture, annotated pathways of DEGs were analyzed using the KEGG database. A total of 90 pathways were enriched in the FKM10 group (Supplementary Figure S1A), among which the most significantly enriched pathways (*p* < 0.05) were ribosome (ko03010); histidine metabolism (ko00340); alanine, aspartate, and glutamate metabolism (ko00250); and ABC transporters (ko02010). The *F. verticillioides* group enriched 243 pathways (Supplementary Figure S1B); nonribosomal peptide structures (ko01054); biosynthesis of amino acids (ko01230); citrate cycle (TCA cycle) (ko00020); 2-oxocarboxylic acid metabolism (ko01210); carbon metabolism (ko01200); valine, leucine, and isoleucine biosynthesis (ko00290); legionellosis (ko05134); sulfur metabolism (ko00920); steroid biosynthesis (ko00100); and nicotinate and nicotinamide metabolism (ko00760) were significantly enriched (*p* < 0.05).

#### Interaction Between Strain FKM10 and *F. verticillioides* Affects the Cell Wall Formation of Each Other

**FIGURE 7:**
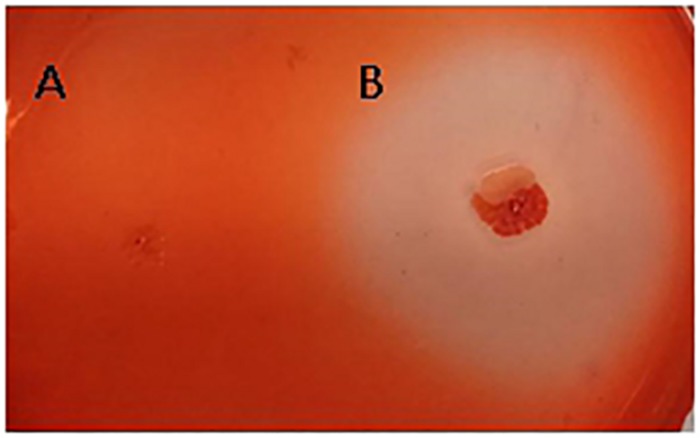
The glucanase activity of strain FKM10. *Escherichia coli* DH5α **(A)** and strain FKM10 **(B)**. Strain FKM10 was inoculated on the glucanase identification medium and incubated at 37°C for 3 days, and then the transparent circle around the colony was observed. *E. coli* DH5α was used as the control.

The fungal cell wall is composed of chitin, 1,3-beta-glucan, 1,6-beta-glucan, mannose, and proteins (Adams, 2004). The characterized hydrolases of the fungal cell wall have chitinase or glucanase activities. In the results of the interaction transcriptome, β-glucanase of strain FKM10 was upregulated, with a 4.56-fold change compared to the results of the strain FKM10 transcriptome (Supplementary Table S2). We also proved that strain FKM10 produced high levels of glucanase (Figure 7), which could hydrolyze the fungal cell wall. We proposed that glucanase might play a role in the process of strain FKM10 inhibiting the growth of *F. verticillioides*.

During the interaction of strain FKM10 and *F. verticillioides*, the synthesis of strain FKM10 cell walls was also reduced due to the lack of nutrients. Cell walls determine the shape of the bacteria and form a protective barrier between the cell and the environment. Peptidoglycan constitutes the cell wall of Gram-positive bacteria, which is covalently linked by anion polymers such as teichoic acid. PhoPR TCS mediates one of the cellular responses of *B. subtilis* to phosphate-limiting conditions (Supplementary Figure S2) (Hulett, 2002), and many of the PhoPR regulator genes have effects, such as alkaline phosphatase (*phoA*, *phoB*, *phoD*) and high-affinity phosphate transporter (*pstSCAB_1_B_2_*) (Bisicchia et al., 2010). Under phosphate-limiting conditions, PhoP ∼ P inhibits *tagAB* transcription and activates *tuaABCDEFGH* transcription, which leads to the conversion of teichoic acid to teichuronic acid to conserve phosphorus for cell metabolism and nucleic acid synthesis (Botella et al., 2014). The cell multiplication rate and cell morphology are also affected (Bisicchia et al., 2010). The expression of *phoD* is also coregulated with that of *tatA*_*d*_ and *tatC*_*d*_ (Pop et al., 2002). In the results of the interaction transcriptome of strain FKM10 (Table 2), the TCS system PhoPR of strain FKM10 sensed phosphorous-limiting signals and phosphorylation, repressed *tagAB* transcription, and activated *tuaABCDEFGH* transcription synthesis of teichuronic acid. Moreover, the phosphate transport operon *pstSACBP* was highly expressed to assist in the transport of phosphate groups.

**TABLE 2:** The expression of genes in phosphate metabolism in *B. velezensis* FKM10.

		**Gene**	**Log2**
**Gene ID**	**Putative function**	**name**	**FC**
**Genes in twin-arginine translocation system**			
ATE50_RS07970	Phosphate ABC transporter%2C permease protein PstA	*pstA*	6.5
ATE50_RS07975	Phosphate ABC transporter ATP-binding protein	*pstB*	6.58
ATE50_RS07980	Phosphate ABC transporter ATP-binding protein	*pstB*	6.9
ATE50_RS07965	Phosphate ABC transporter permease subunit PstC	*pstC*	6.31
ATE50_RS07960	Phosphate-binding protein	*pstS*	7.89
ATE50_RS18015	Alkaline phosphatase	*phoD*	7.96
ATE50_RS06470	DNA-binding response regulator	*phoP*	1.09
ATE50_RS06475	PAS domain-containing sensor histidine kinase	*phoR*	1.07
ATE50_RS14770	Alkaline phosphatase	*phoA*	5.55
ATE50_RS18010	Twin-arginine translocase TatA/TatE family subunit	*tatA*	8.07
ATE50_RS18005	Twin-arginine translocase subunit TatC	*tatC*	5.46
***tua* and *tag* genes**			
ATE50_RS03130	Teichuronic acid biosynthesis protein TuaF	*tuaF*	8.32
ATE50_RS03140	Teichuronic acid biosynthesis glycosyltransferase tuaH	*tuaH*	7.96
ATE50_RS03105	UDP-phosphate *N*-acetylgalactosaminyl-1-phosphate transferase	*tuaA*	7.66
ATE50_RS03110	Lipopolysaccharide biosynthesis protein	*tuaB*	7.77
ATE50_RS03125	Teichuronic acid biosynthesis protein TuaE	*tuaE*	7.75
ATE50_RS03115	Glycosyl transferase family 1	*tuaC*	8.13
ATE50_RS03120	UDP-glucose 6-dehydrogenase	*ugd*	8.21
ATE50_RS03135	Glycosyl transferase	*tuaG*	8.43
ATE50_RS03050	Glycerol-3-phosphate cytidylyltransferase	*tagD*	–1.25
ATE50_RS03040	CDP-glycerol–glycerophosphate glycerophosphotransferase	*tagB*	–1.25
ATE50_RS03070	Teichoic acid ABC transporter ATP-binding protein	*tagH*	–1.3
ATE50_RS03065	Teichoic acid ABC transporter permease	*tagG*	–1.37
ATE50_RS03060	CDP-glycerol–glycerophosphate glycerophosphotransferase	*tagF*	–2.15

#### Protease and Amino Acid Metabolism May Play Key Roles in the Process of Strain FKM10 Inhibiting the Growth of *F. verticillioides*

*Bacillus subtilis* can produce a large amount of extracellular degrading enzymes, such as extracellular proteases (extracellular proteases encoded by the gene *aprE* and bacillus peptidase encoded by the gene *bpr*) (Lopez et al., 2008). Extracellular proteases are essential for bacteria to obtain amino acid substrates. The basic physiological function of extracellular protease is to degrade extracellular high molecular proteins into peptide compounds, which will be further decomposed into oligopeptides and amino acids by peptidase. Therefore, extracellular protease is mainly synthesized and expressed at the end of the logarithmic phase and the early stage of the stable phase, providing more nutrients for the rapid growth of bacteria (Kada et al., 2013). Studies have shown that the overexpression of extracellular protease AprE can significantly increase the valences of bacteriocin A (Barbieri et al., 2016). In the results of intertranscriptomic analysis (Supplementary Table S2), genes of *aprE* and *bpr* of strain FKM10 were upregulated 2.93 and 12.38 times, respectively, and the protein export and bacterial secretion system were upregulated, suggesting that FKM10 might secrete degrading enzymes extracellularly.

Transcriptome analysis of strain FKM10 revealed a large number of DEGs related to amino acid metabolism, including histidine, alanine, aspartate, glutamate, and arginine metabolism. Among these, histidine metabolism genes were significantly downregulated, while arginine metabolism genes were significantly upregulated (Table 3).

**TABLE 3:** Histidine- and arginine-related genes of *B. velezensis* FKM10.

		**Gene**	**Log2**
**Gene ID**	**Putative function**	**name**	**FC**
**Histidine-related genes**			
ATE50_RS09195	Histidinol-phosphate transaminase	hisC	–2.1
ATE50_RS03460	Bifunctional phosphoribosyl-AMP cyclohydrolase/phosphoribosyl-ATP diphosphatase	hisIE	–7.04
ATE50_RS03455	Imidazole glycerol phosphate synthase cyclase subunit	hisF	–6.79
ATE50_RS03450	1-(5-Phosphoribosyl)-5-((5-phosphoribosylamino) methylideneamino) imidazole-4-carboxamide isomerase	hisA	–6.57
ATE50_RS03445	Imidazole glycerol phosphate synthase subunit HisH	hisH	–6.52
ATE50_RS03440	Imidazoleglycerol-phosphate dehydratase	hisB	–6.8
ATE50_RS03435	Histidinol dehydrogenase	hisD	–5.88
ATE50_RS03430	ATP phosphoribosyltransferase	hisG	–6.32
ATE50_RS03425	ATP phosphoribosyltransferase regulatory subunit	hisZ	–6.25
**Arginine related genes**			
ATE50_RS13975	Ornithine carbamoyltransferase	OTC, argF, argI	5.02
ATE50_RS14005	N-acetyl-gamma-glutamyl-phosphate reductase	argC	3.25
ATE50_RS18085	Glutaminase	glsA, GLS	–3
ATE50_RS13995	Acetylglutamate kinase	argB	3.37
ATE50_RS13990	Aspartate aminotransferase family protein	argD	4.03
ATE50_RS06350	Argininosuccinate lyase	argH, ASL	3.65
ATE50_RS06345	Argininosuccinate synthase	argG, ASS1	3.02
ATE50_RS02030	Glutamate dehydrogenase	gudB, rocG	–2.69
ATE50_RS14000	Bifunctional ornithine acetyltransferase/*N*-acetylglutamate synthase	argJ	3.09

#### The Activity of Strain FKM10 Decreased and Spore Synthesis Increased During the Interaction Process of Strain FKM10 and *F. verticillioides*

**FIGURE 8:**
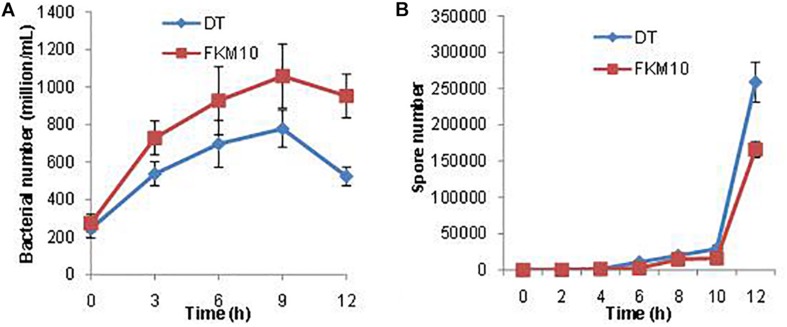
The number of viable cells and spores of strain FKM10 during the interaction process. **(A)** During the interaction process, the number of viable cells of strain FKM10 was determined in the different groups. “FKM10” was the group that was only cultivating strain FKM10. “DT” was the group that was cultivating strain FKM10 with *F. verticillioides*. Samples were taken at different time points, and the diluted samples were applied to LB plates and cultured at 37°C; then the number of single colonies was counted. **(B)** Spore number in the process of interaction. Samples were taken at different time points, and the diluted samples were placed in a constant temperature water bath at 80°C for 15 min to kill the cells. The samples were applied to LB plates and cultured at 37°C, and then the number of single colonies was counted.

During the interaction between strain FKM10 and *F. verticillioides* in the liquid environment, their vitalities were also affected. As seen in the transcriptome data, a large number of ribonucleoprotein complex and flagellar assembly genes in strain FKM10 were downregulated (Supplementary Table S2), which indicated that bacterial activity was decreased. Therefore, the number of living cells in the interaction process was counted (Figure 8A). The results showed that the number of live FKM10 in the interaction group was much lower than that in the group of strain FKM10 only.

Spore formation is reported to initiate by a series of phosphorylation of phosphokinases (KinA, B, C, D, E) and a series of transmission of phosphate groups to Spo0A. The phosphorylated Spo0A initiates the expression of spore-related genes and produces spores (Supplementary Figure S3) (Lopez et al., 2008). KinA is the main kinase of phosphorylation, which is necessary for phosphorylation of Spo0A (Veening et al., 2005). RapA is an aspartamyl phosphatase that has been shown to be critical for maintaining bistable expression of Spo0A (Veening et al., 2005). After the interaction between strain FKM10 and *F. verticillioides*, *spo0A* and spore formation-related genes were upregulated, while those related to spore germination were downregulated (Supplementary Table S4). In addition, more spores were produced by the interaction group than by the group of strain FKM10 (Figure 8B). In addition, *abrB* can negatively regulate spore formation (Shu et al., 2018), and *abrB* is downregulated in the transcriptome results for the interaction group. This result indicates that strain FKM10 was also suppressed during the interaction process and somehow survived by forming a large number of spores.

### Comparative Genomic Analysis of FKM10 to Exhibit the General Features of Species *B. velezensis*

To date, many *B. velezensis* have been reported and 169 genome sequences were obtained. To improve our genetic understanding, comparative genome analysis of strain FKM10 with the other four strains [*B. velezensis* FZB42 (CP000560.1) (Chen et al., 2007), *B. velezensis* JJ-D34 (accession number CP011346) (Jung et al., 2016), *B. velezensis* YJ11-1-4 (accession number CP011347), and *B. velezensis* JS25R (accession number CP009679 and CP009680)] were performed to investigate the similarity of genetic characteristics for this species.

Core and specific genes are used to analyze the functional differences among five genomes (Supplementary Figure S4). The five strains contain 3020 core genes, and this revealed a conversed core genome. Orthologous gene analysis of two strains revealed that strain FKM10 shares fewer orthologous genes with YJ11-1-4 than others. Compared with FZB42, JS25R, and JJ-D34, strain FKM10 possesses the fewest number of specific genes. The specific genes in the strain may benefit their adaptation to different environmental conditions, for example, strain FKM10 in the apple rhizosphere.

The method COGs is useful for systematically compiling the annotated genes and deducing the function of protein families in genomes (Chen et al., 2007). The COGs of the five strains were generated to represent the annotated genes and predict the potential application of the strains (Supplementary Figure S5). The analyzed COG categories of the five genomes are consistent in general. Most genes have been annotated, and the 19% more or less uncharacterized genes are assigned to the S groups. The genes encoding amino acid transport and metabolism, transcription, energy production and conversion, carbohydrate transport and metabolism, and cell wall/membrane/envelope biogenesis account for a larger proportion (each more than 5.8%). The amino acid transport and metabolism genes are the most abundant, accounting for 10.71–10.91% in the five genomes, indicating a better amino acid absorption capacity and response ability to rich amino acids in the living environment. The signal transduction mechanism genes of FKM10 and YJ11-1-4 are enriched compared to FZB42, JJ-D34, and JS25R. FKM10, FZB42, and JS25R have more genes for carbohydrate transport and metabolism. The difference in the COG categories of the five genomes also suggests the genetic diversity and conceivable difference in biological function.

This study enhances our genetic understanding of *B. velezensis* FKM10 for plant-growth promotion. The comparative analysis of the five *B. velezensis* strains at the genetic level also contributes to highlight the shared molecular genetic mechanism of *B. velezensis*. Plant growth-promoting traits of *B. velezensis* species as PGPR are determined by *B. velezensis* FKM10, which will also guide the application of *B. velezensis* to improve crop production as a biocontrol agent or microbial fertilizer.

## Discussion

*Bacillus velezensis* FKM10 was formerly isolated from the apple rhizosphere. In this study, we examined the characteristics of *B. velezensis* FKM10 as a plant growth-promoting rhizobacterium. *B. velezensis* FKM10 can inhibit some fungal pathogens of soil-borne plant diseases effectively, produce siderophores, and degrade proteins. *B. velezensis* FKM10 impacts the growth of *M. hupehensis* Rehd. and the diversity of the rhizosphere microbial community. Moreover, the mechanism of the biocontrol of strain FKM10 toward pathogenic *F. verticillioides* was further illustrated by transcriptome sequencing. Genome comparison further indicated the conservation of the growth promotion and biocontrol of *B. velezensis*.

The results of pot experiments demonstrated that strain FKM10 effectively promoted plant growth. The strain FKM10 could promote the rhizosphere soil nutrients of *M. hupehensis* Rehd., and at the same time, it could change the microbial diversity of rhizosphere soil. The ACE and Chao index can reflect the microbial richness. The Shannon–Weaver and Simpson index can indicate microbial diversity (Zhu et al., 2013). The indexes of the treated group changed, indicating that it can change the bacterial community structure. The microbial biomass and activity act as sensitive indicators for assessing soil quality (Zhang et al., 2016). In addition, differences in soil microbial community structure often indicate differences in nutrient dynamics (Grayston and Prescott, 2005). Therefore, the change in microbial community composition reflects soil nutrient status to some extent (Fierer et al., 2007). In this study, the diversity and richness of the bacterial community structure were increased after applying strain FKM10 compared with the control group, and Planctomycetes was significantly increased. While the richness and diversity of fungal community structure in the treated group were lower than those in the control group, those of Ascomycota were significantly increased, indicating that the number and species of fungi in the soil will decrease after the application of strain FKM10. *B. velezensis* FZB42 was reported to colonize the rhizosphere of lettuce, but not change the composition of rhizosphere bacterial community during 2–5 weeks of plant growth. However, during inoculation with the pathogen and FZB42, the rhizosphere microbial community structure changed (Chowdhury et al., 2013, 2015; Kröber et al., 2014). In our results, the diversity and richness of the bacterial community structure were increased after applying strain FKM10 for a total of 163 days. The *B. velezensis* species might need a long time to change the composition of rhizosphere bacterial community, but the existence of the pathogen could improve the process. Strain FKM10 and FZB42 are both antagonistic bacterium, which can produce a variety of antagonistic substances and inhibit the proliferation of fungi, thereby reducing the richness and diversity of fungi in the soil. Strain FKM10 can improve the ability of inhibiting plant pathogens and indirectly promote the growth of plants (Figures 3, 4). The contents of ammonium nitrogen, nitrate nitrogen, and available iron in the soil were significantly increased, which further indicated that the change in microbial community composition after the application of strain FKM10 had a positive effect on soil nutrients.

To further study the mechanism of *B. velezensis* FKM10 in controlling fungi, *F. verticillioides* was used as an indicator. When strain FKM10 interacted with *F. verticillioides*, strain FKM10 could destroy the cell wall and cell membrane of fungi (Figure 6); at the same time, fungi could also affect the cell wall synthesis of strain FKM10. *B. velezensis* LM2303 was previously reported to damage the cell membrane permeability of *Fusarium graminearumon* by cyclic lipopeptides (fengycin, iturin, surfactin) (Chen et al., 2018). However, the reason that destroyed fungal cell wall and cell membrane is not clear. Moreover, histidine and arginine might also play a positive role in the process of strain FKM10 controlling *F. verticillioides*. On the other hand, strain FKM10 accelerated the formation of spores during the interaction process, and the number of viable cells decreased. The ribonucleoprotein complex and the flagellar assembly gene were all downregulated, indicating that strain FKM10 is also inhibited while antagonizing fungi in a liquid environment.

Some studies have shown that *B. velezensis* can produce a variety of metabolites associated with disease resistance, including antibacterial proteins, lipopeptide antibiotics, polyketides, siderophores, and NH_3_ (Stein, 2005; Chen et al., 2007; Meng et al., 2016; Kim et al., 2017; Adeniji and Babalola, 2019). *B. velezensis* FZB42 was reported to produce high concentrations of siderophores, which could inhibit fungal through depriving essential irons (Rabbee et al., 2019). In this study, strain FKM10 was also able to secrete proteases, such as extracellular protease (*aprE*), bacillus peptidase (*bpr*), and β-glucanase. In the process of interaction, the three genes were all upregulated. It was also reported that Xu et al. (2016) used purified β-1,3-1,4-glucanase (produced by *B. velezensis* ZJ20) to treat three pathogenic fungi, and the mycelium morphology was completely destroyed. The biosynthesis genes for lipopeptide antibiotics, polyketide, and siderophores were also predicted in the genome of strain FKM10 using antiSMASH, including bacilysin, amylocyclicin, difficidin, fengycin, bacillomycin, bacillaene, macrolactin, lantibiotic, and surfactin. However, their biosynthesis genes in strain FKM10 were not upregulated during the interaction process; meanwhile, the biosynthesis genes of difficidin, macrolactin, surfactin, and fengycin were downregulated. These results indicate that some other substances secreted by FKM10 might act as antagonists instead.

In recent years, many strains of *B. velezensis* have been isolated. Their effects on plant growth, resistance to diseases and insect pests, and inducing systemic resistance of plants have been studied. Many strains of *B. velezensis* play important roles as food yield enhancers. To improve our genetic understanding, we analyzed the genome sequence of strain FKM10 to determine its molecular characteristics as PGPR, and further compared with the genomes of four other strains: FZB42, YJ11-1-4, JS25R, and JJ-D34. The gene function analysis of the five *B. velezensis* strains provided deeper insights into understanding the molecular genetic characteristics of this species. The core and specific genes indicated the genetic diversity among the strains (Adeniji and Babalola, 2019). The COG categories of the five genomes analyzed are generally consistent. The results reveal evolutionary conservation of the molecular genetic mechanism of *B. velezensis* strains. The results will further serve as a basis for developing new biocontrol agents or microbial fertilizers by *B. velezensis*.

## Data Availability Statement

The datasets generated for this study can be found in the SRP219725, SRP220076, and SRP221333.

## Author Contributions

YD and BD designed the study. DZ, CW, GQ, and XH performed the laboratory work and analyzed the data. CW and DZ wrote the manuscript. ZM, KL, and BD gave advice on writing the manuscript. YD, BD, ZM, and CW supported the study.

## Conflict of Interest

The authors declare that the research was conducted in the absence of any commercial or financial relationships that could be construed as a potential conflict of interest.

## Footnotes

1 https://github.com/jstjohn/SeqPrep

2 http://www.bowtie-bio.sourceforge.net/bowtie2/manual

3 http://www.biomedsearch.com/nih/RSEM-accurate-transcript-quantification-from/21816040.html

4 http://www.bioconductor.org/packages/2.12/bioc/html/edgeR.html

5 http://www.genome.jp/keg

6 http://kobas.cbi.pku.edu.cn

7 http://www.ncbi.nlm.nih.gov/COG/
