# Supplementary material for: Effects of Bacillus velezensis FKM10 for Promoting the Growth of Malus hupehensis Rehd. and Inhibiting Fusarium verticillioides
Source: Front Microbiol. 2020 Jan 10;10:2889. doi: 10.3389/fmicb.2019.02889 (PMC6965166; doi:10.3389/fmicb.2019.02889)
Supplement: Supplementary file 1 [file Data_Sheet_1.pdf]

# *Supplementary Material*

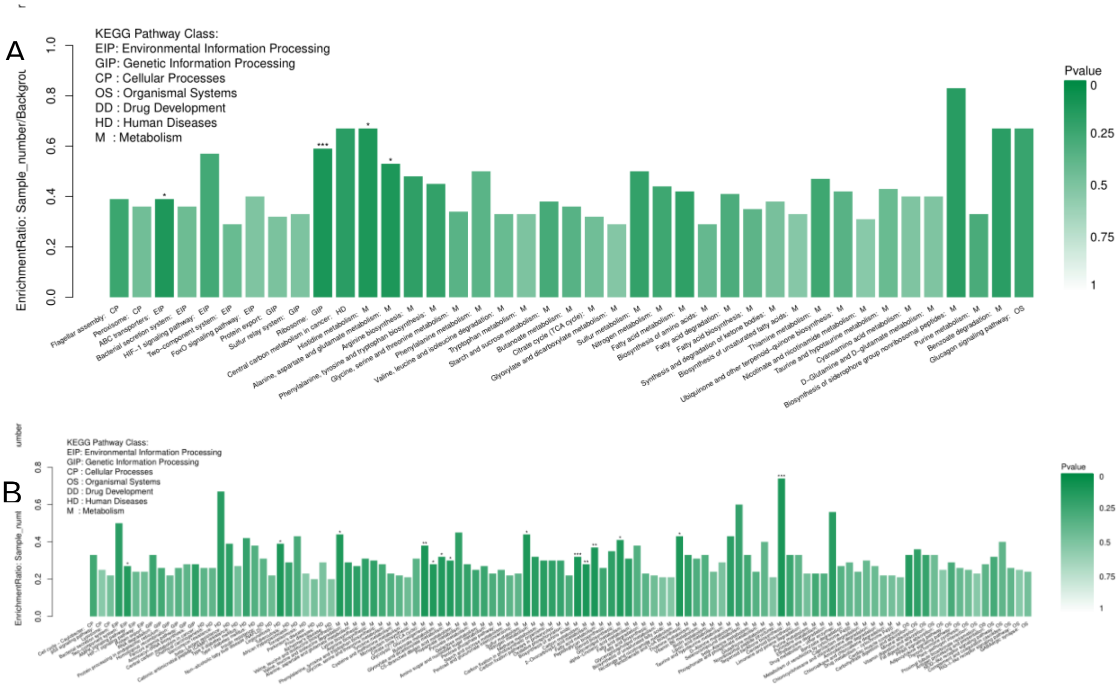


**Supplementary Figure 1.** The KEGG pathway enrichment analysis of the differentially expressed genes of *B. velezensis* (A) and *F. verticillioides* (B). Each column is a path. The abscissa indicates the name and classification of the path. The classification description is shown in the upper left corner. The height of the column indicates the enrichment rate, which is calculated as follows: (EnrichmentRatio=SampleNumber/BackgroundNumber). The color indicates the significance of the enrichment, that is Pvalue. A darker color indicates the more significant enrichment of the pathway. The right color gradient represents the quantitative value of Pvalue.

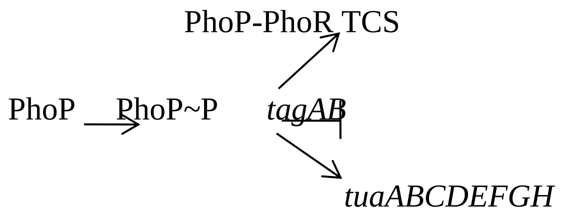


**Supplementary Figure 2.** PhoPR TCS mediatesthe cellular responses in phosphate-limiting conditions. The PhoR kinase phosphorylates PhoP. PhoP~P inhibits *tagAB* and activates *tuaABCDEFGH*.

KinA、B、C、D、E

↓

Spo0F

↓

Spo0B

↓

Spo0A

↓

SpoII→SigF→SigE→SigG→SigK

↓ ↓ ↓

SpoIII Sporulation

**Supplementary Figure 3.** The spore formation process. Phosphorylation of phosphate kinases (KinA, B, C, D, E) involves a series of transfer of phosphate groups from Spo0F and Spo0B to Spo0A, and then the Spo0A phosphorylation initiates the expression of spore-related genes.

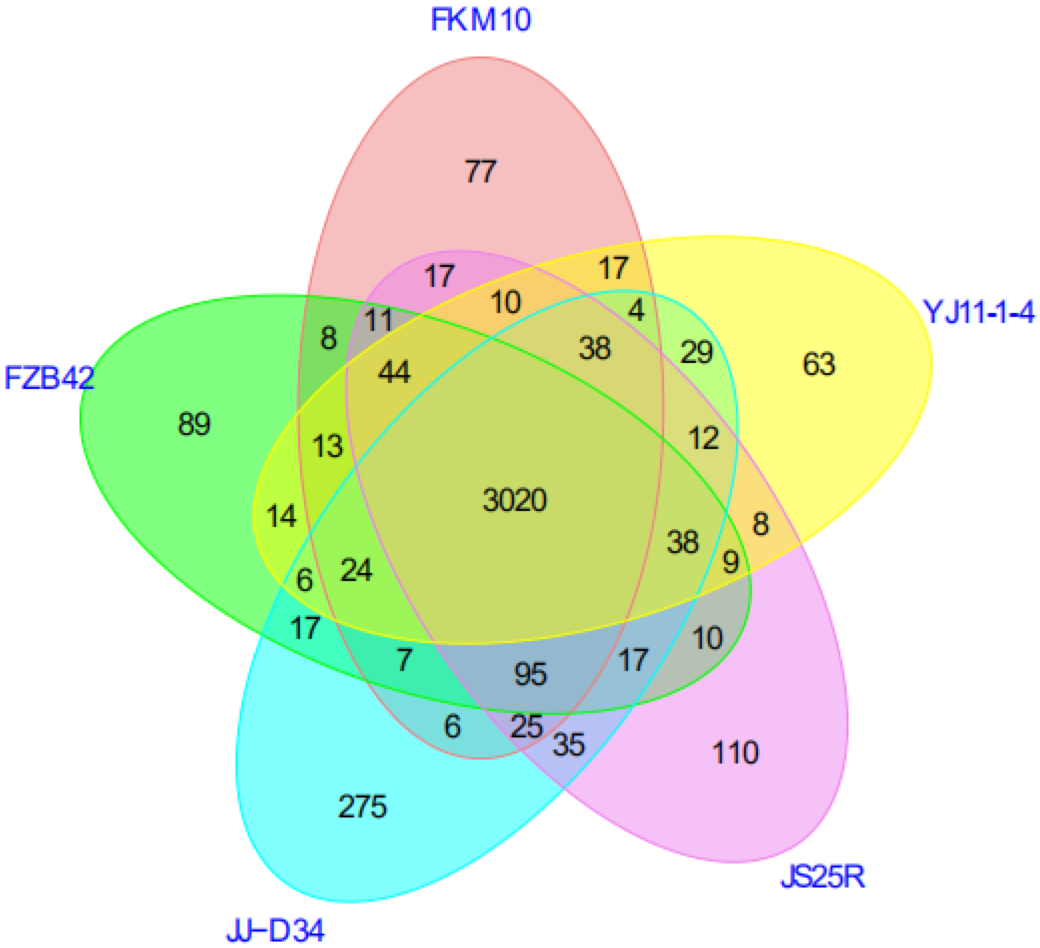


**Supplementary Figure 4.** Core/Pan genes of FKM10 with FZB42, JJ-D34, YJ11-1-4, and JS25R were clustered by the CD-HIT rapid clustering of similar proteins software with a threshold of 50% pairwise identity and 0.7 length difference cutoff in amino acid.

A

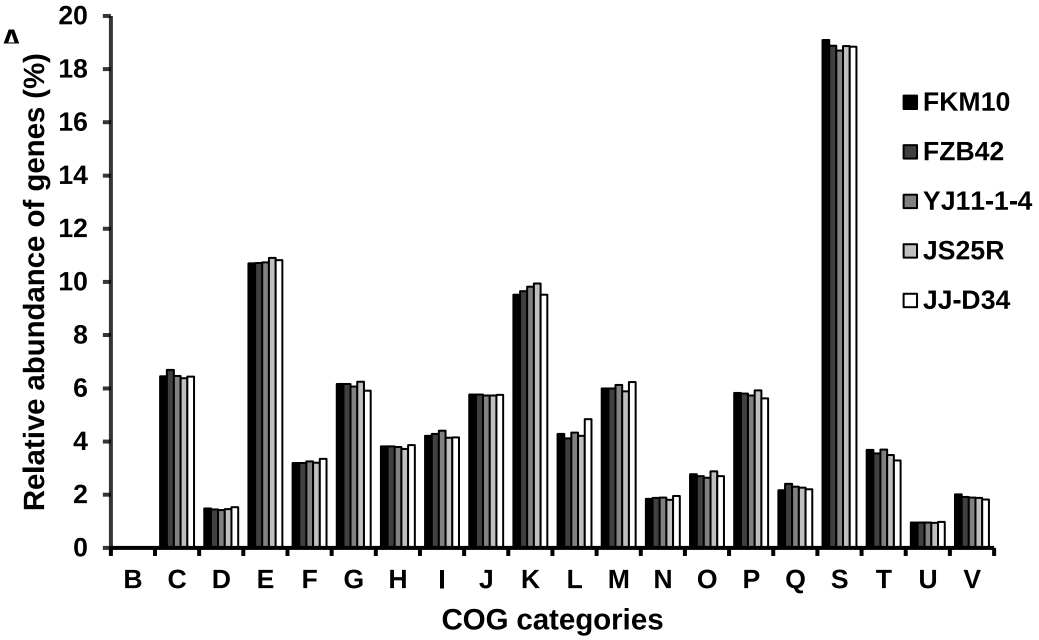


B

| Functional class | Class description |
|---|---|
| B | Chromatin structure and dynamics |
| C | Energy production and conversion |
| D | Cell cycle control, cell division, chromosome partitioning |
| E | Amino acid transport and metabolism |
| F | Nucleotide transport and metabolism |
| G | Carbohydrate transport and metabolism |
| H | Coenzyme transport and metabolism |
| I | Lipid transport and metabolism |

| | |
|---|---|
| J | Translation, ribosomal structure and biogenesis |
| K | Transcription |
| L | Replication, recombination and repair |
| M | Cell wall/membrane/envelope biogenesis |
| N | Cell motility |
| O | Posttranslational modification, protein turnover, chaperones |
| P | Inorganic ion transport and metabolism |
| Q | Secondary metabolites biosynthesis, transport and catabolism |
| S | Function unknown |
| T | Signal transduction mechanisms |
| U | Intracellular trafficking, secretion, and vesicular transport |
| V | Defense mechanisms |

**Supplementary Figure 5.** COG database annotation of FKM10 with FZB42, JJ-D34, YJ11-1-4, and JS25R. A. The relative abundance of genes (%) in the four genomes. B. COG functional classed.
